# Gut and genital tract microbiomes: Dysbiosis and link to gynecological disorders

**DOI:** 10.3389/fcimb.2022.1059825

**Published:** 2022-12-16

**Authors:** Hoda Elkafas, Melinique Walls, Ayman Al-Hendy, Nahed Ismail

**Affiliations:** ^1^ Department of Pharmacology and Toxicology, Egyptian Drug Authority [EDA; formerly The National Organization for Drug Control and Research (NODCAR)], Cairo, Egypt; ^2^ Pritzker School of Medicine, University of Chicago, Chicago, IL, United States; ^3^ Department of Obstetrics and Gynecology, University of Chicago, Chicago, IL, United States; ^4^ Department of Pathology, University of Illinois at Chicago, Chicago, IL, United States

**Keywords:** endometrium, fibroid, PCOS, vaginal microbiome, gut microbiome

## Abstract

Every year, millions of women are affected by genital tract disorders, such as bacterial vaginosis (BV), endometrial cancer, polycystic ovary syndrome (PCOS), endometriosis, and uterine fibroids (UFs). These disorders pose a significant economic burden on healthcare systems and have serious implications for health and fertility outcomes. This review explores the relationships between gut, vaginal, and uterine dysbiosis and the pathogenesis of various diseases of the female genital tract. In recent years, reproductive health clinicians and scientists have focused on the microbiome to investigate its role in the pathogenesis and prevention of such diseases. Recent studies of the gut, vaginal, and uterine microbiomes have identified patterns in bacterial composition and changes across individuals’ lives associated with specific healthy and diseased states, particularly regarding the effects of the estrogen–gut microbiome axis on estrogen-driven disorders (such as endometrial cancer, endometriosis, and UFs) and disorders associated with estrogen deficiency (such as PCOS). Furthermore, this review discusses the contribution of vitamin D deficiency to gut dysbiosis and altered estrogen metabolism as well as how these changes play key roles in the pathogenesis of UFs. More research on the microbiome influences on reproductive health and fertility is vital.

## 1 Introduction

Mounting evidence suggests that the microbiome has a critical role in maintaining human health in almost every organ system—from the skin to the gut to the male and female genital tracts (Rowe et al., 2020). The human microbiome refers to all of the bacteria, viruses, fungi, and archaea living on and within the human body, with the highest number being found in the gut. There is a complex relationship between the human host and its microbial inhabitants. Microbiota piqued scientific interest in many areas of pathological disorders, including tumor biology. Bacteria and their metabolites can control many signaling pathways (e.g., E-cadherin/β-catenin signaling), inducing DNA double-strand breaks and activation of the DNA damage checkpoint pathway, leading to cell death, and modifying cell differentiation, which helps to maintain the homeostasis of the body (Nougayrède et al., 2006; Rubinstein et al., 2013; Eslami-S et al., 2020). Dysbiosis refers to the imbalance of harmful microbes and has been linked to cancer and other diseases (Armstrong et al., 2018; Maynard, 2019; Lu et al., 2021).

**Figure 1:**
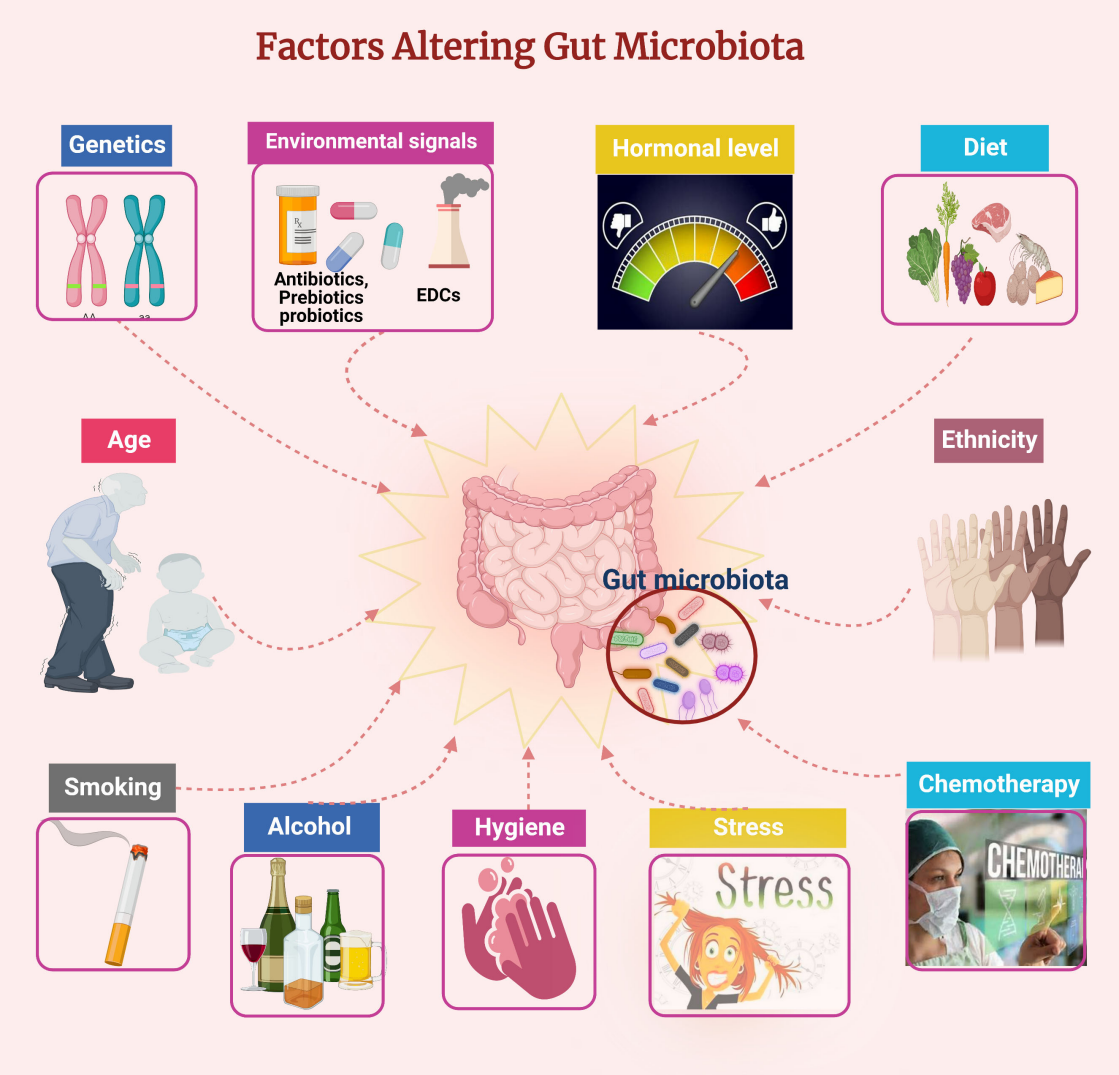
Factors affecting the gut microbiota composition. Figure created using BioRender.com.

Babies acquire their mother’s microbiome at birth through the vaginal canal or face the skin during a Cesarean delivery (Wang et al., 2017). Microbes colonize areas directly exposed to the air or surrounding environment, such as the gastrointestinal tract (including the mouth and stomach), urogenital tract, and skin. Their survival and growth depend on their environment, and each niche has a characteristic collection of microbes. Many factors influence the final bacterial composition within each organ system and among individuals. In particular, the adult microbiome is highly influenced by age, genetics, ethnicity, diet, exercise, chemotherapy, probiotics, prebiotics, smoking, stress, and exposure to endocrine-disrupting chemicals (EDCs), which are well-known factors affecting the populations of different microbes in the gut (**Figure 1**). The microbiome can in turn influence the overall health of the host (Evans et al., 2014; Singh et al., 2017; Hasan and Yang, 2019; Rinninella et al., 2019). The microbiome interacts with the host immune system and is also independently metabolically active, producing factors that alter the pH of the local environment, with consequences for nearby microbes and host tissues.

Recent studies suggest a potential connection between certain gut bacteria and female reproductive tract disorders, such as bacterial vaginosis (BV), cervical and endometrial cancer, polycystic ovary syndrome (PCOS), postmenopausal syndrome, endometriosis, endometritis, and uterine fibroids (UFs) (Mirmonsef et al., 2011; Bodelon et al., 2014; Fuhrman et al., 2014; Guo et al., 2016; Yuan et al., 2018; Łaniewski et al., 2020; Qiu et al., 2022). An investigation into the delicate architecture of the human microbiome may yield insights to improve gynecological health. This review explores the relationships between the gut, vaginal, and uterine dysbiosis and the pathogenesis of various female reproductive tract diseases, such as BV, cervical and endometrial cancer, PCOS, postmenopausal syndrome, endometriosis, endometritis, and UFs. This review also discusses the effect of vitamin D deficiency on the diversity of the gut microbiota and circulated estrogen levels on UF development.

## 2 Female reproductive tract microbiomes

The female reproductive tract comprises the vulva, vagina, cervix, endometrium, fallopian tubes, and ovaries. It harbors a unique microbiome that accounts for approximately 9% of the body’s total microbial population (Moreno and Simon, 2019). The microbiome consists of bacteria (bacteriome), archaea (archaeome), fungi (fungome), viruses (virome), and a few protozoal parasites (Madere & Monaco, 2022). The female reproductive tract microbiome has an essential protective role in supporting vaginal health, reducing the likelihood of preterm birth, and controlling urogenital infections such as BV, yeast infections, and sexually transmitted infections (STIs), including human immunodeficiency virus (HIV) (Punzón-Jiménez and Labarta, 2021). Studies on the female reproductive tract microbiome have mainly focused on determining the bacterial composition and functions (Franasiak and Scott, 2015). At least five bacterial community groupings are represented in the female reproductive tract, distinguishable by the abundance of *Lactobacillus* spp. and the existence of more diverse anaerobes (Smith & Ravel, 2017; Madere and Monaco, 2022).

### Upper versus lower reproductive tract microbiomes

The upper reproductive tract comprises the fallopian tubes, ovaries, and uterus, including the endocervix, and the lower tract consists of the ectocervix and vagina. The microbiome of the female genital tract varies between the lower and upper tracts, with the lower tract having a bacterial burden of 100–10,000-fold more than the upper tract (Chen et al., 2017; Baker et al., 2018). In 2011, Ravel et al. (2011) described five community state types (CSTs) in the vagina of healthy women, determined mainly by the dominant species of *Lactobacillus* and pH value, with CSTs I, II, III, and V involving dominant *L. crispatus*, *L. gasseri*, *L. iners*, and *L. jensenii*, respectively. CST IV has a low abundance of *Lactobacillus* and higher abundances of specific anaerobic genera (*Atopobium*, *Dialister*, *Gardnerella*, *Prevotella*, and *Sneathia*) (Ravel et al., 2011). *Lactobacillus* (i.e., *L. crispatus* and *L. iners*) was abundant in 92% of cervical specimens from healthy women (Ravel et al., 2011). Another study confirmed that the microbiome is strongly altered from the lower to the upper genital tract, with decreased *Lactobacillus* in the upper genital tract compared to that in the lower genital tract (Chen et al., 2017). The female reproductive tract microbiome is not fixed but changes with age, physiological conditions, lifestyle, and environmental factors.

#### 2.1.1 Vaginal microbiome

The composition of the vaginal microbiome is important in reproductive health. *Lactobacillus* is one of the main bacteria colonizing the healthy vagina. Its production of lactic acid as a metabolic bioproduct inhibits the overgrowth of potentially pathogenic yeast, bacteria, and viruses, thus limiting phylogenetic diversity in the vaginal microbiome. Epidemiological research has linked the abundance of *Lactobacillus* to ethnicity, with Caucasian/Asian women having high levels of *Lactobacillus* compared to African/Hispanic women (Gupta et al., 2019). The mean pH varies from 4.0 ± 0.3 for CST I to 5.3 ± 0.6 for CST IV (Ravel et al., 2011; Punzón-Jiménez and Labarta, 2021). In the presence of estrogen, vaginal epithelial cells accumulate glycogen that can be metabolized by *Lactobacillus* into lactic acid (Miller et al., 2016), resulting in decreased vaginal pH and less colonization by pathogenic bacteria such as *Chlamydia trachomatis*, *Neisseria gonorrhoeae*, and *Trichomonas vaginalis* (Chen et al., 2017; Champer et al., 2018; Chambers et al., 2021).

The implications of vaginal dysbiosis are represented by the condition of BV. BV is a common cause of abnormal vaginal discharge, and sex hormones may play a role in BV pathogenesis. Estrogen increases vaginal epithelial activity, resulting in a condensed epithelium with more glycogen deposited in the superficial cells (Punzón-Jiménez and Labarta, 2021). This vaginal acidity is vital in preventing the overgrowth of BV-associated bacteria. Decreased estrogen is associated with vaginal dysbiosis, involving the replacement of regular hydrogen peroxide- and lactic acid-producing *Lactobacillus* in the vagina with a high abundance of anaerobic bacteria such as *Gardnerella*, *Mycoplasma*, and *Prevotella*, which increases the vaginal pH to >4.5 (Punzón-Jiménez and Labarta, 2021). Anaerobic bacteria associated with BV include *Gardnerella vaginalis*, which has virulence factors that allow it to bind to the host epithelium and generate a biofilm community that contributes to BV symptoms (Schwebke et al., 2014). Vaginal dysbiosis is made more likely by contraceptives, hormonal changes, obesity, and hysterectomy (Gupta et al., 2019).

#### 2.1.2 Endometrial microbiome

The endometrium plays a critical role in the reproductive process in premenopausal women. It is associated with complex physiological changes influenced by estrogen and progesterone during the menstrual cycle and pregnancy. It is also affected by factors such as prostaglandins, integrins, angiogenesis, and immune-modulating cytokines. The first study on the bacterial composition of the uterus reported that real-time quantitative PCR identified 95% of 58 patients undergoing hysterectomy as having a uterine microbiota, with the most common species being *L. iners* (in 45%) and *Prevotella* (in 33%) (Mitchell et al., 2015). Several subsequent studies have found that *Lactobacillus* is the most prominent genus in the endometrium, with *Lactobacillus* being identified in both endometrial fluid and endometrial biopsies (Franasiak et al., 2016; Moreno et al., 2016; Miles et al., 2017; Moreno and Simon, 2019; Kiecka et al., 2021). It is unclear whether *Lactobacillus* is an actual endometrial colonizer or whether it ascended from the vagina. These results were consistent with another study on 70 women, with additional findings of *Streptococcus*, *Staphylococcus*, *Corynebacterium*, and *Bifidobacterium* (Tao et al., 2017). Moreno and Simon (2019) reported that among 35 endometrial samples from fertile healthy women, *Lactobacillus* was the most abundant genus (71.1%), followed by *Gardnerella*, *Bifidobacterium*, *Streptococcus*, and *Prevotella* (12.6%, 3.7%, 3.2%, and 0.9%, respectively) (Moreno & Simon, 2019). A low percentage of *Lactobacillus* in the female vaginal sample is less likely to have a successful embryo implantation, with an interplay between the uterine microenvironment and the host’s immune system (Moreno et al., 2022). This involves increased pro-inflammatory cytokine production in endometrial cells, including interleukin (IL)-1α, IL-1β, IL-17α, and tumor necrosis factor (TNF)-α, due to the estrogen-dependent nature of endometrial cell cytokine production (Sobstyl et al., 2022). However, other groups reported that *Proteobacteria*, *Firmicutes*, and *Bacteroidetes* were prevalent in endometrial samples, while *Lactobacillus* spp. was only found in a low percentage of endometrial samples (Verstraelen et al., 2016; Chen et al., 2017; Burac, 2018; Liu et al., 2019). Future endometrial microbiota studies need to focus on the effect of the microbiota on the female genital tract to potentially enhance the treatment of gynecological disorders. Although several studies have highlighted the predominance of *Lactobacillus* in the uterine microbiota, there remains uncertainty about the bacterial composition related to the healthy endometrium.

#### 2.1.3 Fallopian tube and ovarian microbiomes

The fallopian tubes and ovaries harbor highly variable microbial species among different women. Additionally, a study found high intraindividual variability between fallopian tubes and ovaries, with *Bacteroides*, *Corynebacterium*, *Lactobacillus*, *Coprococcus*, and *Hymenobacter* being found in fallopian tube fluid specimens, and *Lactobacillus*, *Corynebacterium*, *Escherichia*, and *Blautia* being found in ovarian fluid specimens (Miles et al., 2017). Chen et al. (2017) reported that the fallopian tubes and ovaries had lower *Lactobacillus* than the vagina and cervix. In contrast, Pelzer et al. (2011) reported that fallopian tube specimens lacked *Lactobacillus*, but they did have at least some bacterial species. In addition, Pelzer et al. (2011) showed that, regarding the ovarian bacterial community, *L. iners*, *Actinomyces* spp., *Corynebacterium aurimucosum*, *Fusobacterium* spp., *Prevotella*, and *Staphylococcus* spp. were more dominant in the left ovarian follicular fluid than the right. Fallopian tubes and ovaries harbor a mixture of bacteria that thrive in mildly alkaline states, contrasting with the acidity of the vagina (Punzón-Jiménez and Labarta, 2021).

## 3 Gut microbiome and link to estrogen metabolism

### 3.1. Overview of gut microbiome

The gut microbiome is a complex ecosystem of microbes acting upon and changing in response to the host environment (Gilbert et al., 2018; Chambers et al., 2021). The gut microbiome is dominated by five bacterial phyla: phylum Firmicutes (*Clostridium*, *Lactobacillus*, *Eubacterium, Ruminococcus*, *Butyrivibrio*, *Anaerostipes*, *Roseburia*, *Faecalibacterium*, etc.; Gram-positive species), phylum Bacteroidetes (*Bacteroides*, *Porphyromonas*, *Prevotella*, etc.; Gram-negative species), phylum Proteobacteria (*Enterobacteriaceae*, etc.; Gram-negative species), phylum Actinobacteria (*Bifidobacterium*; Gram-negative species), and phylum Verrucomicrobia (*Akkermansia*, etc.; Gram-negative species) (Doaa et al., 2016). Regarding the gut microbiota, almost 90% are Firmicutes and Bacteroidetes, while the rest are Actinobacteria and Proteobacteria. Verrucomicrobia is the least abundant (Yurtdaş and Akdevelioğlu, 2020; Sędzikowska and Szablewski, 2021). The gut microbiome’s structure is developed from birth and modulated by delivery mode and the maternal skin and gut microbiomes (Dominguez-Bello et al., 2010).

The gut microbiota influences the intestinal milieu, which acts on other organs and pathways. A healthy gut microbiota improves digestion, metabolism, and immune response; the symbiotic relationship between the host’s gut and the microbiota that maintains homeostasis is known as normobiosis (Rea et al., 2018).

Gut microbiomes are responsible for metabolizing estrogen; estrogens are metabolized by microbe-secreted β-glucuronidase, which converts estrogen from a conjugated form to a deconjugated form (Plottel and Blaser, 2011; Williams et al., 2020). Deconjugated and unbound (active and free) estrogens circulate in the bloodstream and bind to estrogen receptors alpha (ERα) and beta (ERβ) (Baker et al., 2017). Phytoestrogens are similarly metabolized and act *via* ERα and ERβ (Baker et al., 2017). The binding of estrogens and phytoestrogens to ERs initiates downstream signaling (Rietjens et al., 2017). This ultimately alters physiological conditioning, affecting reproductive health and neural development (Dewi et al., 2012).

### 3.2 Estrobolome and endobolome

The estrobolome refers to the gut microbiota genes involved in the metabolism of estrogen (Plottel and Blaser, 2011; Graham et al., 2021), while the endobolome refers to the gut microbiota genes and pathways involved in the metabolism of steroid hormones (including estrogen) and EDCs (Aguilera et al., 2020). EDCs have been defined by the US Environmental Protection Agency as “exogenous chemicals that interfere with the synthesis, secretion, metabolism, binding activity, or elimination of natural blood-borne hormones that are present in the body and are responsible for homeostasis, reproduction, and developmental function” (Diamanti-Kandarakis et al., 2009; Zoeller et al., 2016; Aguilera et al., 2020). EDCs include natural chemicals found in nature (phytoestrogens, genistein, and coumestrol) and artificial chemicals utilized in industrial solvents/lubricants and their by-products [polychlorinated biphenyls (PCBs), polybrominated biphenyls (PBBs), and dioxins], plasticizers (phthalates), pesticides [methoxychlor, chlorpyrifos, and dichlorodiphenyltrichloroethane (DDT)], antibacterials (triclosan), fungicides (vinclozolin), diethylstilbestrol (DES), and bisphenol A (BPA) (Diamanti-Kandarakis et al., 2009; Darbre, 2017; Aguilera et al., 2020). EDC exposure can cause marked gut dysbiosis, which can lead to disorders in many organ systems (Rosenfeld, 2017).

## 4 Gut dysbiosis and gynecological disorders

**Figure 2:**
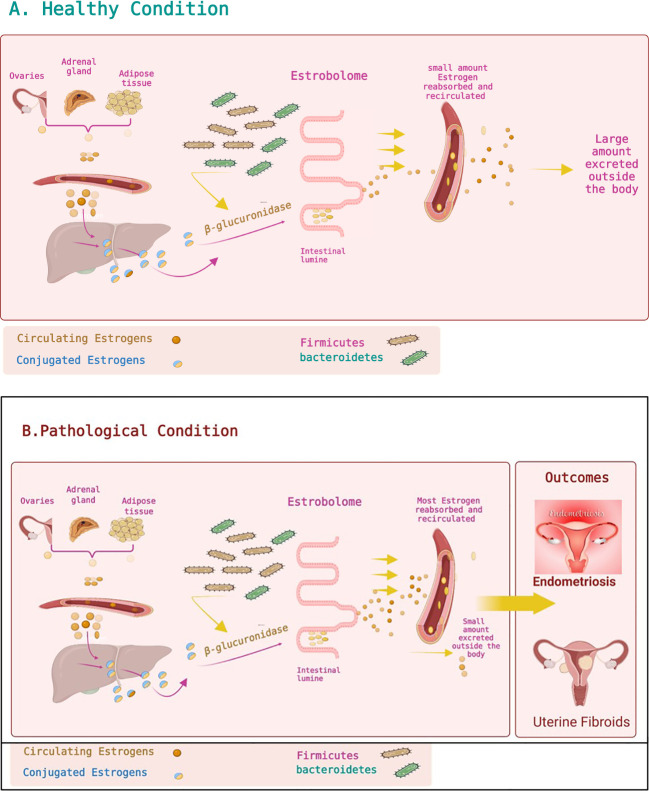
Estrobolome and estrogen metabolism. **(A)** Healthy condition. Estrogens are mainly produced by the ovaries, adipose tissue, and adrenal glands and distributed *via* the bloodstream in a free or protein-bound state. Estrogens and their metabolites can then be conjugated in the liver. Conjugated estrogens are excreted from the liver after metabolic transformation to water-soluble molecules and passed in the bile to the gut. The conjugated estrogens eliminated in the bile can be deconjugated by gut bacteria that have β-glucuronidase activity, leading to estrogen reabsorption into the bloodstream. Circulating estrogens impact target tissues, including the uterus, stimulating cell proliferation. The estrobolome affects estrogen elimination and circulation by regulating the enterohepatic circulation of estrogens. **(B)** Pathological condition. Altering the estrobolome by increasing the Firmicutes*/*Bacteroidetes ratio leads to more deconjugated estrogen and more circulating estrogen, impacting specific organs and contributing to multiple diseases such as endometriosis and uterine fibroids. Figures are created using BioRender.com.

Estrogen–gut microbiome relations have physiological and clinical outcomes. Dysbiosis and a drop in gut microbiota abundance affect the estrobolome, leading to broad-range disorders (Baker et al., 2017). Dysbiosis and inflammation reduce the gut microbiota and reduce the β-glucuronidase activity. This reduction in the function of β-glucuronidase is followed by free estrogen reduction in the blood circulation. This is followed by altering estrogen receptor activations, leading to a hypoestrogenic pathologic status: obesity and metabolic syndrome, including PCOS. Furthermore, the estrobolome causes a hyperestrogenic pathologic status *via* a rise in the abundance of β-glucuronidase-producing bacteria, i.e., an increased Firmicutes/Bacteroidetes ratio, which boosts levels of free circulating estrogens and increases binding to ERα and ERβ, causing diseases such as endometrial cancer, breast cancer, endometriosis, and UFs to force disorders such as endometriosis and cancer (Plottel and Blaser, 2011; Dewi et al., 2012; Baker et al., 2017) (**Figures 2A, B**). Apparently, the gut microbiome regulates estrogen metabolism and implicates estrogen-related disorders (Flores et al., 2012; Sommer and Bäckhed, 2013; Liu et al., 2017). Moreover, EDC exposure in the developmental stage is linked to the development of UFs later in life. This is due to an altered estrogen balance and inhibition of DNA damage repair machinery, which lead to conversion of myometrium stem cells to tumor-initialing stem cells (Prusinski Fernung et al., 2018; Elkafas et al., 2019).

### 4.1 Polycystic ovary syndrome

PCOS is a hormonal disease caused by an imbalance between estrogen and androgen. Tremellen and Pearce (2012) studied the role of gut dysbiosis in PCOS development due to poor diet. Dysbiosis increases gut mucosal permeability, resulting in the entry of lipopolysaccharide from Gram-negative colonic bacteria into the circulation. The resultant activation of the immune system interrupts insulin receptor function, increasing serum insulin levels, raising the ovaries’ production of androgens, and interfering with regular follicle production (Tremellen and Pearce, 2012). Studies have shown that lower circulating unconjugated estrogen and increased testosterone in PCOS patients may alter the gut microbiome (Tremellen and Pearce, 2012; Liu et al., 2017). Regarding the role of gut microbiota on PCOS, studies done by Guo et al. (2016) in 2016 showed that treatment of female Sprague–Dawley rats with letrozole (a nonsteroidal aromatase inhibitor) promoted PCOS and gut dysbiosis and altered the sex hormone levels, estrous cycles, and ovarian morphology. *Lactobacillus*, *Ruminococcus*, and *Clostridium* were downregulated while *Prevotella* was upregulated in PCOS rats compared to control rats (Guo et al., 2016). The same group showed that after treating PCOS rats with *Lactobacillus* and fecal microbiota transplantation (FMT) from healthy rats, the estrous cycles were enhanced in all animals in the FMT study group and 75% of rats in the *Lactobacillus* transplantation group had diminished androgen biosynthesis and normalized ovarian morphologies. The bacterial composition of gut microbiota was fixed in both FMT and *Lactobacillus*-treated groups, raising *Lactobacillus* and *Clostridium* and reducing *Prevotella* (Guo et al., 2016). A study indicated that the gut dysbiosis related to PCOS involved decreased bacterial diversity and an altered Firmicutes/Bacteroidetes ratio (Thackray, 2019). The role of the lower genital tract microbiota in PCOS has also been investigated. A study of vaginal and cervical biopsies showed that there were increased pathogens (such as *Gardnerella vaginalis*, *Mycoplasma hominis*, and *Prevotella*) and reduced *Lactobacillus* in both the vagina and cervix in PCOS patients compared to healthy controls (Tu et al., 2020). These findings were consistent with another study showing increased *Mycoplasma* and *Prevotella* and decreased *L. crispatus* in vaginal and cervical samples from PCOS patients compared to healthy controls (Hong et al., 2020). Thus, the estrobolome may affect sex hormone-driven disorders involving estrogen, such as PCOS.

### 4.2 Postmenopausal syndrome

Menopause is the permanent stop of menstrual cycle resulting in the loss of folliculogenesis and reduction in estrogen levels resulting in modifications impacting all body functions (Dalal and Agarwal, 2015). The gut microbiota may be important in regulating estrogen levels and estrogen metabolism during and after menopause. In postmenopausal women, gut microbiota diversity is linked to higher estrogen metabolite levels relative to parent estrogen levels in urine (Fuhrman et al., 2014). The gut microbiota also metabolizes estrogen-like compounds in food (such as soy isoflavones) and stimulates specific bacterial growth. Supplementation with soy isoflavones in postmenopausal women increases *Bifidobacterium* and reduces unclassified *Clostridiaceae* (Qi et al., 2021). *Bifidobacterium* has a beneficial role in stimulating absorption and immunity and inhibiting intestinal infection, while *Clostridiaceae* is involved in inflammatory diseases and is associated with obesity. These effects suggest that this host–microbiome estrogen-related interaction contributes to a broad spectrum of pathways influencing women’s health and illness (Nakatsu et al., 2014; Qi et al., 2021).

### 4.3 Cervical and endometrial cancer

During dysbiosis, invasion of the vagina by many anaerobic bacteria as the abundance of *Lactobacillus* decreases leads to inflammatory reactions that promote tumor growth, thus causing diseases such as cervical intraepithelial neoplasia, which can develop into cervical cancer (Champer et al., 2018). Endometrial cancer is the fourth most common cancer in women and the fifth most common cause of cancer death in the United States (Brooks et al., 2019). In endometrial cancer, estrogens are essential for increasing inflammation by upregulating the production of pro-inflammatory mediators such as IL-6 and TNF-α, which can in turn synergistically upregulate the expression of enzymes involved in ovarian steroidogenesis (aromatase, 17β-hydroxysteroid dehydrogenase, and estrone sulfatase), thus producing a feedback loop (Wallace et al., 2010; Borella et al., 2021). Moreover, high estrogen can change the vaginal microbiome, indirectly sustaining the development of endometrial cancer by interfering with the gut and vaginal microbiomes (Nakamura et al., 2019; Borella et al., 2021). A cross-sectional study analyzed the microbiomes of the vagina, cervix, fallopian tubes, and ovaries and found that the bacterial taxa in the genital tract of patients with endometrial cancer are characterized by the predominance of Firmicutes (*Anaerostipes*, *Dialister*, *Peptoniphilus*, *Ruminococcus*, and *Anaerotruncus*), Spirochaetes (such as *Treponema*), Actinobacteria (*Atopobium*), Bacteroidetes (*Bacteroides* and *Porphyromonas*), and Proteobacteria (*Arthrospira*) (Walther-António et al., 2016; Borella et al., 2021). Another study examined the influence of *Atopobium vaginae* and *Porphyromonas somera* on endometrial cells *in vitro*, revealing that these bacteria increase the release of inflammatory cytokines and chemokines (IL-1α, IL-1β, IL-17α, and TNF-α) by endometrial cells, indicating their role in maintaining inflammation and in neoplastic initiation (Caselli et al., 2019). This was supported by another study demonstrating a link between genital tract dysbiosis, high inflammatory cytokine expression, and endometrial cancer (Lu et al., 2021).

### 4.4 Endometriosis and endometritis

Endometriosis is characterized by chronic inflammation in which the endometriotic cells promote the synthesis of ILs, TNF-α, and vascular endothelial growth factor (VEGF), generating the conditions for tumor transformation (Kokcu, 2011; Jiang et al., 2021). Endometritis is an infectious and inflammatory disorder of the endometrium (Kitaya et al., 2018).

Estrogen promotes epithelial cell proliferation throughout the female genital tract, and estrogen dysregulation is associated with proliferative disorders such as endometriosis (Zhang et al., 2009; Anderson, 2019). Endometriosis is common in premenopausal women and is associated with a hyperproliferative state secondary to elevated estrogen levels (Laschke and Menger, 2016). In 2002, Bailey and Coe (2002) showed that monkeys with endometriosis had a low abundance of *Lactobacillus* and a high abundance of Gram-negative bacteria. Recently, research has shown that endometriosis is accompanied by gut microbial dysbiosis; however, the exact findings vary among studies. For instance, Ata et al. (2019) showed that patients with moderate-to-severe endometriosis (n = 14) have more *Shigella/Escherichia* in their colon than healthy individuals (n = 14). In contrast, Shan et al. (2021) showed that patients with stage 3–4 endometriosis (n = 12) had lower gut microbiota alpha diversity and a higher Firmicutes/Bacteroidetes ratio than healthy participants. A recent large study by Svensson et al. (2021) on stool samples reported that the abundance of 12 bacteria in the classes *Bacilli*, *Bacteroidia*, *Clostridia*, *Coriobacteriia*, and *Gammaproteobacteria* strongly varied between endometriosis patients (n = 66) and healthy individuals (n = 198). The discrepancy between these studies necessitates more extensive studies of how endometriosis stage, age, race, medical history, medication use, and diet affect the bacteria involved in endometriosis (Chadchan et al., 2022). An animal study reported that at 42 days after mice were intraperitoneally injected with endometrial tissue to trigger endometriosis, there was a significant elevation in the Firmicutes/Bacteroidetes ratio between the endometriotic and control mice (Yuan et al., 2018).

**Figure 3:**
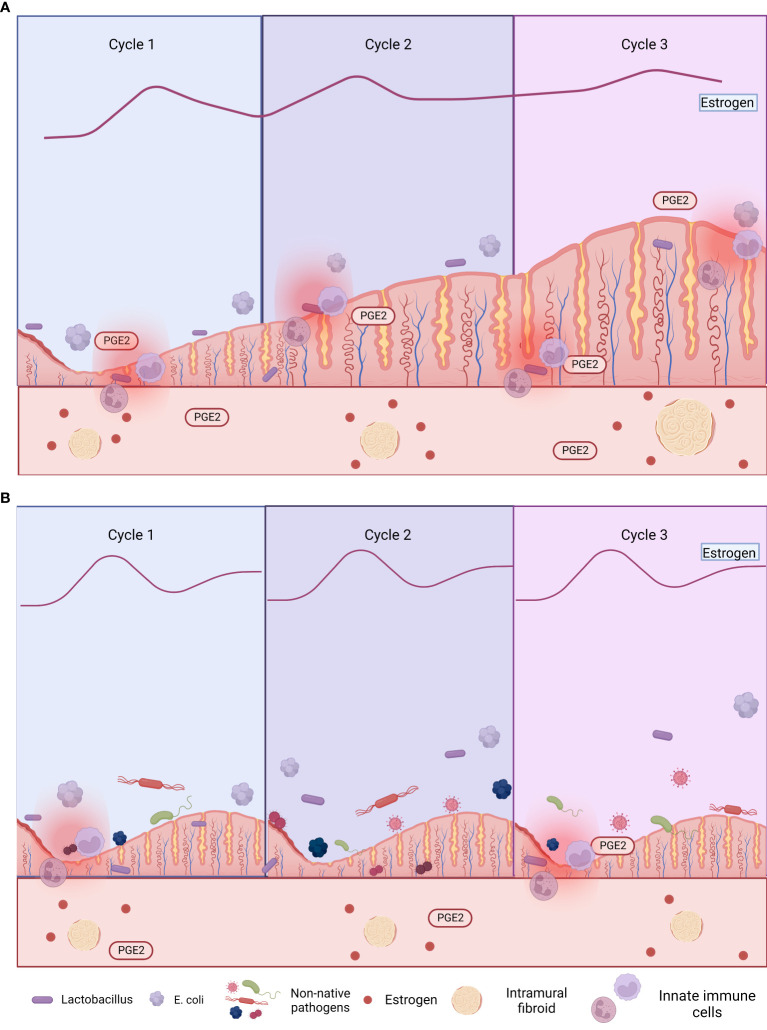
Comorbid endometriosis and uterine fibroids that are **(A)** untreated and **(B)** treated with gonadotropin-releasing hormone agonist (GnRHa). Excess estrogen exposure is associated with both endometriosis and uterine fibroids (UFs), so estrogen is a target of multiple treatments (e.g., GnRHa and aromatase inhibitors). However, these treatments have local immunosuppressive effects, increasing susceptibility to uterine dysbiosis and subsequent endometriosis (Khan et al., 2014; Qu et al., 2021). Figures are created using BioRender.com.

Several studies have examined the relationship between hormone dysregulation, the uterine microbiota, and gynecological disorders such as endometriosis (Khan et al., 2014; Walther-António et al., 2016; Hernandes et al., 2020; Schreurs et al., 2021). Investigated the intrauterine microbiome colonization and occurrence of endometritis in women with endometriosis and controls (fertile women with no evidence of endometriosis), without significant clinical differences between the two groups. A subset of women from both groups was treated with a gonadotropin-releasing hormone agonist (GnRHa) for 4–6 months before collecting uterine and vaginal samples. Both groups, with or without endometriosis, had 16 types of anaerobic and aerobic bacteria and yeast colonizing the vagina. However, notably, subclinical uterus infection was more common in endometriosis patients than controls and much more common in those treated with GnRHa than in those who did not receive GnRHa (Khan et al., 2014). This was attributed to the presumption that GnRHa alters the intrauterine environment, thus increasing uterine infection in women with endometriosis. There was a significant increase in the diagnosis of endometritis in the GnRHa-treated women than untreated women, with or without endometriosis, due to the increased endometrial colonization rate caused by GnRHa. Higher abundances of multiple bacteria (including *Staphylococcaceae*, *Moraxellaceae*, *Lactobacillacae*, *Streptococcaceae*, and *Enterobacteriaceae*) were detected in endometrial swabs from GnRHa-treated women than untreated women, with or without endometriosis. *Streptococcaceae* and *Staphylococcaceae* increased and *Lactobacillacae* decreased in GnRHa-treated compared to untreated endometriosis patients, indicating that low estrogen due to GnRHa increases intrauterine infection, thus causing endometrial dysbiosis (Khan et al., 2014). Microbes from the vaginal area can move to and colonize the endometrium (where they are usually exposed to a different environment) (Khan et al., 2014). The increased intrauterine colonization in endometriosis patients was hypothesized to be attributable to increased prostaglandin E2 (PGE2), which caused immunosuppression that enabled bacterial survival and replication. In support of this hypothesis, PGE2-mediated immunosuppression of lymphocytes is higher in women with endometriosis than those without endometriosis (Khan et al., 2014). Notably, the presence of pathogenic bacteria (such as *Escherichia coli*) and enhanced bacterial colonization in women with endometriosis caused subclinical endometritis and further promoted endometriosis severity and/or complications (Khan et al., 2014). As sex steroids (such as estrogen) modulate the number of antimicrobial agents [such as defensins and secretory leukocyte protease inhibitor (SLPI)] in the endometrium and fallopian tubes (**Figure 3A**) (Khan et al., 2014), altering estrogen levels can lead to intrauterine microbial infection and/or tissue inflammation, leading to dysbiosis (**Figure 3B**). These studies concluded that altering estrogen levels triggers inflammatory responses in the endometrial tissues, leading to microbial colonization of the uterus, endometriosis, and endometritis (Khan et al., 2014).

### 4.5 Uterine fibroids

UFs or leiomyomas are benign monoclonal tumors of the uterus derived from uterine smooth muscle (myometrium). They affect >70% of women of reproductive age worldwide, especially women of color (Wise and Laughlin-Tommaso, 2016; Al-Hendy et al., 2017). UFs significantly impact patients’ health, fertility, and economic status. In the United States, the annual financial burden of UF treatment is estimated to have reached $34.4 billion (Giuliani et al., 2020). There is a wide range of invasive and noninvasive treatment options, from short-term pharmacotherapies to laparoscopic or open myomectomy or hysterectomy (Istre, 2008; Yang et al., 2022). The treatment depends on the tumor size, tumor location, age, and reproductive plans. Epidemiological studies indicate that African American women develop UFs more frequently and at an earlier age, and the tumors tend to be more aggressive, growing larger and causing more significant symptoms than in Caucasian women (Al-Hendy and Salama, 2006; Eltoukhi et al., 2014; Stewart et al., 2016).

A characteristic feature of UFs is their dependence on the ovarian steroid hormones estrogen and progesterone (Elkafas et al., 2017). These hormones stimulate complex paracrine signals, which allow UF cells to secrete mitogenic stimuli and thereby influence adjacent immature UF cells, thus providing UF cells with undifferentiated cells that can support tumor growth (Ono et al., 2013; Reis et al., 2016). Estradiol increases the sensitivity of tissues to progesterone by increasing the availability of progesterone receptors (PRs) (McGuire et al., 1977; Mote et al., 2002; Elkafas et al., 2017). Selective PR modulators (SPRMs) are synthetic steroid ligands designed to modulate the PR site in a tissue-specific way; binding of SPRMs to PRs can cause agonistic effects, antagonistic effects [e.g., they can compete with agonists or enhance the effects of co-repressors to cause antagonistic effects (Islam et al., 2020)], or mixed effects. They can induce receptor dimerization and DNA binding and coordinate with co-activators and/or co-repressors (Madauss et al., 2007). Asoprisnil and ulipristal acetate are SPRMs, while raloxifene is a selective ER modulator that suppresses UF growth (ElKafas et al., 2018). Research using these compounds can help to study estrogen- and progesterone-dependent gynecological disorders.

In recent years, high-throughput sequencing has been used to study the gut microbiome diversity in UF patients. A case–control study examined the differences in follicle-stimulating hormone (FSH), estradiol (E2), and anti-Mullerian hormone (AMH) before and after transabdominal hysterectomy for UF patients and the effect of transabdominal hysterectomy on the gut microbiome (Wang et al., 2020). The results indicated that FSH increased after surgery, while E2 and AMH decreased. The ovaries are involved in hormone secretion and ovulation, and the study concluded that transabdominal hysterectomy altered ovary function, leading to decreased E2 and AMH and increased FSH. This decreased estrogen affected the gut microbiome diversity, with increased Proteobacteria and Firmicutes and decreased Bacteroidetes (Wang et al., 2020; Yang et al., 2022).

## 5 Vitamin D deficiency and its link to gut dysbiosis and uterine fibroids

Diet and nutrition are key factors that maintain microbiome homeostasis in the body. Vitamin D is an essential nutrient for microbiome homeostasis. Vitamin D receptors (VDRs) are widely expressed in the intestines, immune system (Liu et al., 2018), uterus, and liver (Zinser et al., 2005; Clark and Mach, 2016). Vitamin D levels not only affect the gut microbiome but also impact the airway microbiome (Robles-Vera et al., 2019).

According to the current UF pathogenesis theory, vitamin D is one of the most significant contributing factors to the development of this disease (Sharan et al., 2011; Ciebiera et al., 2018). Reduced serum vitamin D is a risk factor for the development of UFs (Brakta et al., 2015; Elkafas et al., 2021). Epidemiological studies show that African American women have lower vitamin D levels than Caucasian women (Laughlin et al., 2010). Among infertile patients with various ethnicities, UF patients had lower vitamin D than women without UFs, suggesting that vitamin D deficiency in UF patients can occur independently of ethnicity (Sabry et al., 2013). Vitamin D level is inversely proportional to UF volume, regardless of ethnicity (Sabry et al., 2013). A study on the effect of low VDR levels on the pathogenesis of UFs indicated that 60% of women with UFs had low VDR levels (Halder et al., 2013a). The authors noted that inadequate or loss of VDR is a risk factor for UF pathogenesis (Halder et al., 2013b).

The effect of vitamin D on gut microbiota diversity has been examined in several studies. A study reported that vitamin D supplementation increased the overall diversity of the gut microbiota, which involved increasing Bacteroidetes and decreasing Firmicutes (thereby improving the Firmicutes/Bacteroidetes ratio) and increasing the phyla *Verrucomicrobia* and *Actinobacteria* (Singh et al., 2020). *Akkermansia muciniphila*, which belongs to the phylum Verrucomicrobia, assists in maintaining host intestinal homeostasis by transforming mucin into beneficial by-products, thus maintaining gut homeostasis. The abundance of *A. muciniphila* is negatively associated with body mass, inflammation, and metabolic syndrome (Singh et al., 2020). In 2015, Wu et al. (2015) placed fecal communities into two enterotypes based on *Prevotella* and *Bacteroides* levels. They observed that vitamin D intake increased *Bacteroides* and decreased *Prevotella* (Wu et al., 2015), which was a previously reported benefit of vitamin D (Singh et al., 2020). It was noted in the studies by Wu et al. (2015) and Singh et al. (2020) that vitamin D is essential in maintaining gut microbiome homeostasis, and deficiency causes gut dysbiosis.

Vitamin D aids in gut microbiome homeostasis by activating VDRs in the proximal colon. VDRs are transcription factors that regulate the expression of over 1,000 host genes (Singh et al., 2020). These genes are involved in gut microbiome homeostasis, inflammation, prevention of pathogen invasion, and commensal bacterial colonization. The interactions between VDR signaling and the gut microbiome affect host immune responses and inflammation (Singh et al., 2019). Vitamin D has been considered a treatment option for UFs. Arjeh et al. (2020) conducted a randomized controlled trial on the effect of oral vitamin D supplementation on UF growth. They concluded that as vitamin D deficiency causes dysbiosis that leads to the pathogenesis of UFs, vitamin D supplementation could be a treatment option for UFs (Arjeh et al., 2020). The 12-week randomized controlled trial in UF patients showed no significant difference in the volume of fibroids in the oral vitamin D group despite an increase in the placebo group (Arjeh et al., 2020). It was concluded that vitamin D inhibits UF cell proliferation *via* its indirect effects on the gut microbiome (Arjeh et al., 2020).

VDRs inhibit bacteria-induced pro-inflammatory nuclear factor NF-κB activity in the gut (Wu et al., 2015). Supplementation with 25 µg/kg vitamin D in pregnant mice before a 100-µg/kg lipopolysaccharide injection activated VDR signaling, inhibited the pro-inflammatory NF-κB p65 pathway, and decreased the expression of the inflammatory cytokines TNF-α, IL-Iβ, and IL-6 (Clark and Mach, 2016). VDRs maintain the intestinal barrier function by decreasing intestinal permeability, dysbiosis, inflammation, and lack of immune tolerance in the gut (Cantorna et al., 2014). Accordingly, VDR-knockout mice are more sensitive to lipopolysaccharide-induced endotoxemia, with increased expression of inflammatory cytokines [e.g., TNF-α, interferon IFN-γ, IL-1a, IL-1β, IL-21, and IL-10] and more weight loss, bleeding, ulceration, septic shock, and death compared to control mice (Froicu and Cantorna, 2007). In autoimmune patients, vitamin D3 may directly interact with the gut microbiota and reduce dysbiosis (Ananthakrishnan et al., 2014). In a cohort of 3,188 inflammatory bowel disease patients, higher plasma vitamin D3 (27.1 ng/ml) was associated with a remarkably decreased risk of *Clostridium difficile* infection (Ananthakrishnan et al., 2014). A study of multiple sclerosis (MS) patients revealed that oral administration of 5,000 IU of vitamin D3 daily for 90 days increased *Akkermansia*, which stimulates immune tolerance, and increased *Faecalibacterium* and *Coprococcus*, which both generate butyrate, a short-chain fatty acid with anti-inflammatory, antiproliferative, and apoptotic effects (Cantarel et al., 2015). A case series of seven relapsing-remitting MS (RRMS) patients demonstrated that vitamin D supplementation increased *Enterobacteria* in both healthy controls and RRMS patients, with shifts in Firmicutes, Actinobacteria, and Proteobacteria in the RRMS patients (Mielcarz and Kasper, 2015). The vitamin D/VDR complex increases innate immune cells’ chemotactic and phagocytic capabilities and activates the transcription of antimicrobial peptides in several cell types, including colonic cells (Lagishetty et al., 2010). Vitamin D induced the expression of antibacterial peptides cathelicidin (hCAP18) and human β-defensin-2 (HBD-2), which are expressed in breast milk and the intestinal mucosa and are involved in innate immunity, promoting numerous functions such as chemotaxis of inflammatory immune cells and microbicidal action (Wampach et al., 2017).

**Figure 4:**
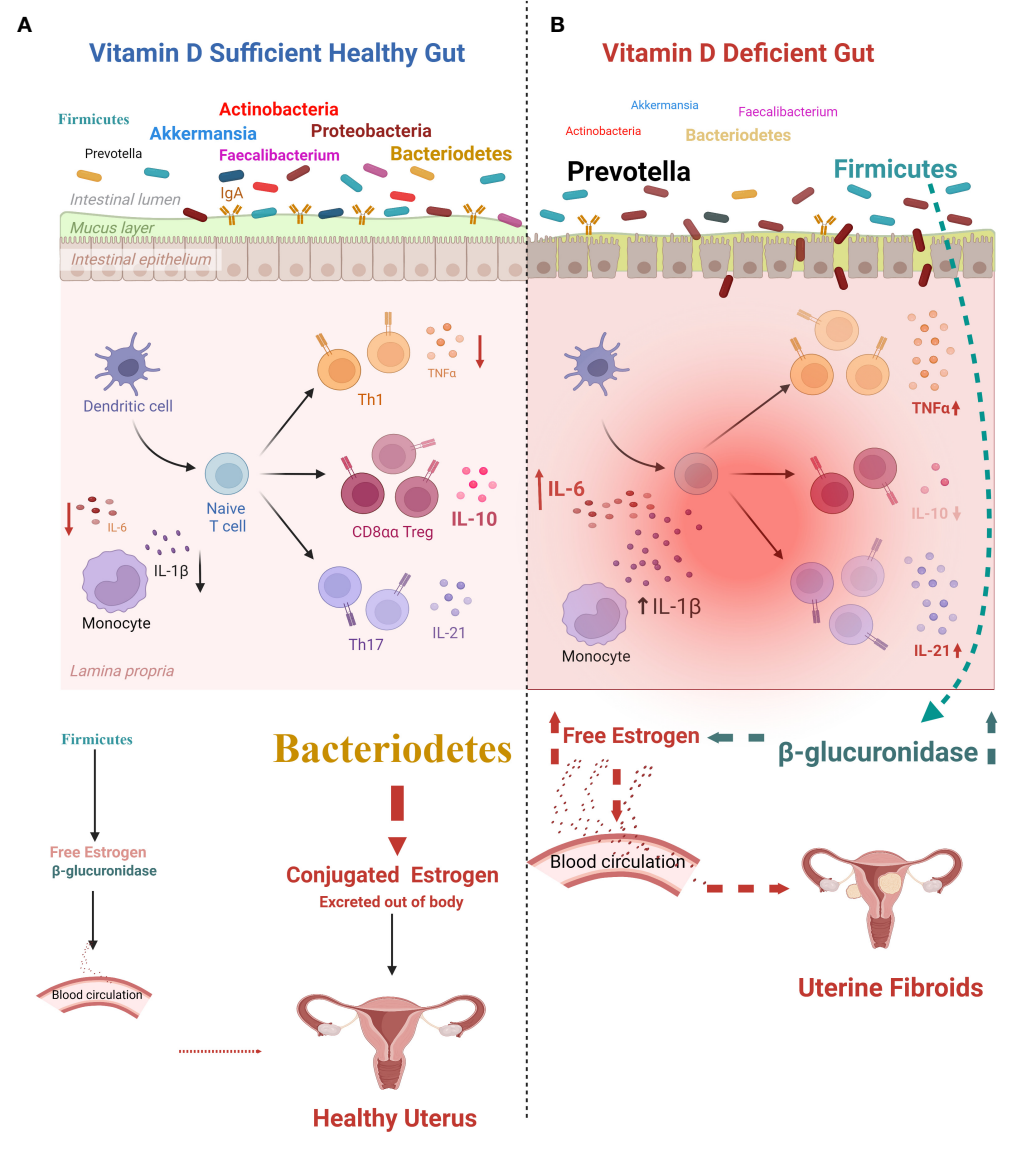
Effects of vitamin D on the gut and estrogen metabolism. **(A)** Effect of vitamin D sufficiency: healthy gut. Dominant gut bacteria (including Bacteroidetes) are anti-inflammatory, preserve gut homeostasis, and maintain estrogen metabolite levels, decreasing the incidence of uterine diseases. **(B)** Effect of vitamin D deficiency: pathological gut. Lower vitamin D level leads to gut inflammation with increased release of interleukin (IL)-1β, IL-6, tumor necrosis factor (TNF)-α, and IL-21, which causes gut dysbiosis (involving an increase in the Firmicutes/Bacteroidetes ratio) followed by an increase in the release of unbound estrogen into the circulation. This increases the incidence of uterine fibroids (UFs). Figure created using BioRender.com.

Mice fed a vitamin D-deficient diet exhibited a pre-fibroid status in the myometrium marked by increased expression of sex steroid receptors and proliferation-related genes, high DNA damage, and fibrosis progression (ElHusseini et al., 2018). There was also higher inflammation and immunosuppression in the vitamin D-deficient mice (due to increased regulatory T cells in the myometrium) compared to the controls (ElHusseini et al., 2018). The same research group reported a preliminary study on Eker rats of the effect of early-life exposure to EDCs on myometrium stem cells. They found a significant increase in inflammatory mediators (IL-6, IL-17, IL-1β, IL-1α, TNF-α, and NF-κB) produced by the exposed MMSCs compared to unexposed myometrial stem cells (MMSCs) (Elkafas et al., 2019). Vitamin D reversed these inflammatory mediators (lowering IL-6, IL-17, IL-1β, IL-1α, TNF-α, and NF-κB) and enhanced the anti-inflammatory effect by increasing IL-4 and IL-10 (Elkafas et al., 2019). Concluding that, the gut microbiome may be involved in inflammation *via* the endobolome, and vitamin D deficiency decreases *Akkermansia*, which reduces immune tolerance, and increases Firmicutes, which increases free estrogen. Thus, vitamin D is an essential nutrient that maintains the homeostasis of the gut microbiome (**Figure 4**).

## 6 Conclusions

Conditions such as BV, cervical and endometrial cancer, PCOS, postmenopausal syndrome, endometriosis, endometritis, UFs, and bacterial infections negatively affect women’s reproductive health and quality of life. The human microbiota plays a vital role in maintaining women’s overall health and preventing these conditions. The human microbiome’s composition and function are critical in understanding the pathogenesis of these diseases. Several studies over the last 20 years have demonstrated a strong association between the microbiome and chronic gynecological disorders. These disorders have come to be better understood by investigating associated disturbances in the uterine, gut, and vaginal microbiome, and future studies may involve therapeutic manipulation of the human microbiome, as in the case of vitamin D supplementation for UF prevention and treatment. Further research is required to fully understand the role of the microbiome and its interactions with the human host in the prevention, development, and treatment of genital tract disorders.

## Author contributions

NI and HE designed the scope and organization of the review. HE conducted literature reviews, constructed figures, and contributed to the writing of most of the manuscript. MW edited and summarized some sections, create one figure and write the abstract. NI and AA-H supervised the writing and critically edited and reviewed the complete manuscript and figures. All authors contributed to the article and approved the submitted version.
