# Erratum: Gut and genital tract microbiomes: dysbiosis and link to gynecological disorders

**DOI:** 10.3389/fcimb.2023.1211349

**Published:** 2023-05-12

**Authors:** Frontiers Production Office

**Affiliations:** Frontiers Media SA, Lausanne, Switzerland

**Keywords:** endometrium, fibroid, PCOS, vaginal microbiome, gut microbiome

Incorrect Author Name

Due to a production error, an author’s name was incorrectly spelled as “Melinique Wall”. The correct spelling is “Melinique Walls”. The publisher apologizes for this mistake.

The original version of this article has been updated.

